# *In-vivo* impact of common cosmetic preservative systems in full formulation on the skin microbiome

**DOI:** 10.1371/journal.pone.0254172

**Published:** 2021-07-07

**Authors:** Barry Murphy, Michael Hoptroff, David Arnold, Richard Eccles, Stuart Campbell-Lee

**Affiliations:** 1 Unilever Research & Development, Port Sunlight, Bebington, Wirral, England, United Kingdom; 2 Institute of Infection, Veterinary, and Ecological Sciences, University of Liverpool, Liverpool, England, United Kingdom; Skin Research Institute Singapore, SINGAPORE

## Abstract

Preservatives play an essentially role in ensuring that cosmetic formulations remain safe for use via control of microbial contamination. Commonly used preservatives include organic acids, alcohols and phenols and these play an essential role in controlling the growth of bacteria, fungi and moulds in substrates that can potentially act as a rich food source for microbial contaminants. Whilst the activity of these compounds is clear, both *in vitro* and in formulation, little information exists on the potential impact that common preservative systems, in full formulation, have on the skin’s resident microbiome. Dysbiosis of the skin’s microbiome has been associated with a number of cosmetic conditions but there currently are no *in vivo* studies investigating the potential for preservative ingredients, when included in personal care formulations under normal use conditions, to impact the cutaneous microbiome. Here we present an analysis of four *in vivo* studies that examine the impact of different preservation systems in full formulation, in different products formats, with varying durations of application. This work demonstrates that despite the antimicrobial efficacy of the preservatives *in vitro*, the skin microbiome is not impacted by preservative containing products *in vivo*.

## Introduction

The human skin microbiome, the collection of bacteria, viruses and fungi that inhabit the human skin surface and invaginations has become a research topic of fundamental interest. Skin is the largest epithelial surface that is colonised by microbes [1–3], and its microbial inhabitants are believed to play an important role in the maintenance of healthy skin [4,5]. Microbial composition of the skin varies across body sites driven by the nutrients available in characteristic dry (arm), moist (axilla) and sebaceous (scalp) sites across the body [6]. This site-specific community composition means that in reality, the skin microbiome can more appropriately be considered a consortium of distinct microbiomes [7].

It is well documented that numerous members of the skin microbiome play a crucial role in the production of various metabolites that have an impact on skin health including free fatty acids from *Cutibacterium acnes* [8,9], antimicrobial peptides and phenol soluble modulins from *Staphylococcus epidermidis* [10,11] and other staphylococcal species [12,13]. An abundance of these organisms is commonly associated with healthy skin. Conversely, the presence of skin disease is commonly accompanied by a shift in microbiome composition to one taxonomically or functionally distinct from an individual’s baseline healthy state. Common conditions associated with microbiome community alterations include acne [14], atopic skin [15], dandruff [16] and axillary odour [17]. In some instances, a causal microbiome variation has been unequivocally proven [18] whereas in others conditions a complete understanding of the complex multifaceted interactions between microbes and human skin has yet to be completely characterised [19].

To control cosmetic conditions the use of personal care products has become common place. These products attempt to alleviate symptoms through the use of ingredients including skin moisturisers and topical antimicrobials [20,21]. However, despite the use of these products, in the absence of disease flares, variations in seasons, environmental conditions or perturbations in the structure or integrity of the skin barrier, the temporal stability of the skin microbiome has been shown to be robust [22,23]. This suggests a degree of community resilience to the skin microbiome, an essential ability of the community to respond to disturbance and return to its previous state, both structurally and functionally [24]. Ensuring community resilience is not compromised by the application of cosmetic products has been identified as a key consumer safety parameter [25].

Cosmetics are produced in a non-sterile but hygienically controlled environment however inadvertent contamination may occur. In addition, microbial challenge can be common place during consumer use [26]. Cosmetic ingredients can act as nutrient sources to facilitate the growth of contaminating microorganism under the appropriate physiochemical conditions. This contamination can range from Gram-negative and Gram-positive bacteria, yeasts and fungi, many of which are opportunistic pathogens which can cause serious infection and illness [26,27]. Therefore, every manufacturer of cosmetics has a responsibility to ensure the microbiological safety of its products for the intended use lifespan [28]. Under EU cosmetics regulations, each cosmetic product placed on the market should have its own Product Information File (PIF) which captures details on the microbiological quality of the raw materials and the finished product [29].

Cosmetic manufactures use approved preservative systems to maintain product quality and protect against the growth of spoilage and pathogenic microorganisms [30]. The preservatives system must have a broad spectrum of activity and be compatible with the product ingredients as well as being stable over the shelf-life and intended usage time [31]. To achieve this, a combination of preservatives and formulation hurdles are used to gain a broad spectrum of activity and reduce the necessary concentration of single actives [32]. For example, the use of certain ingredients and formulation hurdle benefits such as pH control and reduced water activity can be used to improve the innate robustness of the product or to potentiate antimicrobial activity [27].

The most common antimicrobial preservatives can be divided into a number of groups according to their chemical structure and functional groups. These include, organic acids, alcohols, phenols, aldehydes, and formaldehyde donors, isothiazolinones, biguanides, quaternary ammonium compounds, nitrogen compounds, heavy metal derivatives, and inorganic compounds [27]. Each of these preservative groups will have a different mode of action and spectrum of activity under the correct concentration and formulation properties. The use of certain preservatives may be limited, or the concentration restricted depending on the type of product and the area for application. For example, rinse-off products such as shampoo will typically have fewer restrictions compared to leave-on products such as skin moisturiser, which have prolonged skin contact [33].

The activity of preservatives has been examined *in vitro* using standard minimum inhibitory concentration tests [34,35], which can provide insights into antimicrobial performance in the neat product. However, there is limited information on the impact of different preservative systems on the skin microbiome under *in vivo* conditions. Only through the use of *in vivo* analysis are realistic effects of product usage, dilution, cutaneous substantivity and the ability of the microbiome to respond to perturbations by seeding the skin from protected invaginations faithfully represented [23]. Where previous analysis sought to investigate the impact of preservative ingredients on skin bacteria *ex vivo* [36], this analysis sought to investigate the impact of cosmetic formulations containing four different commonly used preservative systems on the skin microbiome of healthy adult females *in vivo*.

Leg skin microbiome samples were collected from each subject at baseline and after final product application and assessment on the impact of the preservative containing products was carried out using standard microbiome analysis including taxonomic and diversity analysis.

## Materials and methods

### Study populations and test materials

Key inclusion and exclusion criteria for each study population (A-D, corresponding to preservative system A-D) included females between 18–55 years of age in general good health with intact skin free of disease. A complete list of inclusion and exclusion criteria is outlined in S1 Table. The compositions of the preservative systems examined are outlined in Table 1 and a list of the associated formulation ingredients can be found in S2 Table.

**Table 1:** Summary information on the different preservation systems, product formats, and product application regime used in the subsequent analysis.

Preservation System Name	Preservation System Composition	Format	Timepoints Assessed	Application
**A**	• Glydant Plus (IPBC/DMDM) [0.26%] • Tetrasodium EDTA [0.05%]	Wash off shower gel	Baseline (pre use) and 24 hours post use.	Once
**B**	• Phenoxyethanol [0.6%] • Tetrasodium EDTA [0.05%] • IPBC [0.007%]	Wash off shower gel	Baseline (pre use) and 24 hours post use.	Once
**C**	• Glydant Plus (IPBC/DMDM) [0.26%] • Tetrasodium EDTA [0.05%]	Wash off shower gel	Baseline (pre use) and following 2 weeks of use.	Once daily for 2 weeks
**D**	• Methylparaben (0.2%) • Propylparaben (0.1%) • Phenoxyethanol (0.4%)	Leave on lotion	Baseline (pre use) and following 5 weeks of use.	Twice daily for 5 weeks

Each preservative containing formulation was applied to 15 subjects per study with varying frequency of application and study duration. The study location and relevant population metadata can be seen in Table 2.

**Table 2:** Summary information for cohorts of subject used for subsequent analysis.

Preservation System	Study Location	Sex	Age Range	Average Age
**A**	Canada	Female	21–63	51.7
**B**	USA	Female	19–65	39.5
**C**	USA	Female	21–50	33.2
**D**	UK	Female	21–51	37.6

Subjects enrolled in the studies were not subjected to a conditioning phase and were permitted to continue using their standard cleansers in advance of the study. This was to retain the natural variation seen in a subject’s skin microbiome as a result of their current hygiene routine and environment. All subjects were required not to bathe or apply cosmetics for a minimum of 12 hours before sampling. For studies where products C and D were applied post application samples were taken 12–18 hours after last product application.

### Sample selection

Samples were selected for this cohort analysis from 4 separate intervention studies carried out to examine the impact of cosmetic formulations on the adult leg skin microbiome. Written informed consent for all studies was obtained from all enrolled individuals. The study protocols were reviewed and approved by the appropriate independent ethics committees, Study A: Institutional Review Board Services, Study B and C: Gallatin Institutional Review Board, Study D: Reading Independent Ethics Committee. The methods were carried out in accordance with the principles of the Declaration of Helsinki and principles of Good Clinical Practice as applicable to clinical studies on cosmetics. Sample collection, shipping and processing of samples for all studies were carried out in an identical fashion minimising any potential bias as a result of sample collection methodologies or data processing variations. No adverse events were reported for any of the studies in question and all subjects enrolled in the study maintained good skin condition throughout the study.

### Microbiome sample collection and processing

Buffer washes were collected using a sterile Teflon sampling ring with a 3.5cm internal diameter (total diameter 5cm and height 3.5cm) using the method previously described [37]. The ring was placed in the sampling site and held firmly in place by a second operator. Using a digital pipette and barrier (filter) pipette tip, 2.0ml of buffer wash solution (sterile phosphate-buffered saline pH7.9 containing 0.1% Triton X-100) was placed into the sampling ring and the skin surface gently agitated for one minute with a sterile Teflon rod (with rounded, smooth ends). The sampling fluid was collected using a sterile disposable pipette and placed into a sterile centrifuge tube. The sampling procedure was repeated with a further 2.0ml aliquot of buffer wash material and both aliquots pooled. Samples were placed on ice during the collection process and then stored at -80°C prior to DNA extraction. Shipment of samples from all studies prior to extraction was carried out on dry ice with appropriate temperature logging.

### DNA extraction

Samples were defrosted and concentrated by centrifugation (10mins/13,000rpm, Eppendorf 5810R, Germany), supernatant removed, and the cells resuspended in 500μl of sterile TE buffer (10 mM Tris-HCl; 1 mM EDTA, pH 7.4). The cell suspension was transferred to a 96-well Lysing Matrix Plate B (MP Biomedicals). Addition of 3ul of Ready-Lyse lysozyme (Epicentre, 250U/ul) was followed by incubation with agitation at 300rpm, 37°C for 18 hours. Following incubation, a bead-beating step was performed using a Tissue Lyser (Qiagen, Germany) for 3 minutes at 20 Hz. An off-board lysis was performed by incubating the samples at 68°C for 15 minutes in the presence of Proteinase K, Carrier RNA, ATL and ACL buffer in a Qiagen S-plate following manufacturer guidelines. Post-incubation, the samples were processed using the QIAamp UCP DNA Micro kit according to manufacturer instructions (56204, Qiagen). Extracted DNA was frozen prior to 16S rRNA gene library preparation.

### 16S rRNA library preparation and sequencing

Oligonucleotide primers targeting the V1-V2 hypervariable region of the 16S rRNA gene were selected. PCR was carried out using the following primers,

U28F: 5’-ACACTCTTTCCCTACACGACGCTCTTCCGATCTNNNNNAGAGTTTGATCMTGGCTCA G-3’

U338R: 5’-GTGACTGGAGTTCAGACGTGTGCTCTTCCGATCTTGCTGCCTCCCGTAGGAGT-3’

PCR primers were modified version of the standard 28F and 338R primers which contain additional recognition sequences to facilitate nested PCR to add Illumina sequencing adapters and index sequences to resulting amplicons using methods described previously [38].

A second round PCR incorporated Illumina adapters containing indexes (i5 and i7) for sample identification utilising eight forward primers and twelve reverse primers each of which contained a separate barcode allowing up to 96 different combinations. General sequences of the primers are illustrated below with the variable 8 bp barcode underlined and amplification carried out as previously described [38].

N501f 5′AATGATACGGCGACCACCGAGATCTACAC*TAGATCGC*ACACTCTTTCCCTACACGACGCT3′

N701r 5′CAAGCAGAAGACGGCATACGAGAT*TCGCCTTA*GTGACTGGAGTTCAGACGTGTGCTC3′.

### Informatics processing

All steps were performed using the QIIME2 microbiome analysis tool suite [39] version 2019.1. The paired end sequences were imported into QIIME2 format, then denoised using DADA2 [40]. The primer sequence regions were removed during denoising by setting DADA2’s forward and reverse read trim parameters to the length of the forward and reverse primers, respectively. A complete list of software parameters and versions can be found in S3 and S4 Tables. Rooted and unrooted phylogenetic trees were generated for the ASVs (Amplicon Sequence Variants) using the QIIME2 phylogeny align-to-tree-mafft-fasttree workflow. Taxonomic assignments were generated by comparing ASVs against a BLAST database composed of the HOMD, HOMD extended and Greengenes sequences (HOMDEXTGG version 14.51) described in [41]. Taxonomic classification was performed as previously described [42] at 99% identity across 98% of the read length. Visualization and plotting of resulting data was carried out using the QIIME2 suite and JMP v 14.1 [43].

### Statistical analysis

All statistical analysis were carried out using the QIIME2 microbiome analysis tool suite [39] version 2019.1. Within sample group diversity (alpha) changes were estimated and tested using non-parametric approaches. A signed rank test for changes across time-points for each treatment that accounts for paired differences within subjects. Kruskal Wallis tests were used for pairwise treatment comparisons. Between group diversity (beta) was assessed visually using principal co-ordinate ordination plots for key metric distance matrices, Bray-Curtis(semi-metric), Jaccard, weighted and unweighted Unifrac [44]. Statistical inference was achieved using permutation analysis of variance (PERMANOVA). Taxonomic differences in mean relative abundance were assessed using ANCOM (analysis of compositions) [45] to access differences within treatments across timepoints.

## Results

### Taxonomy

**Fig 1:**
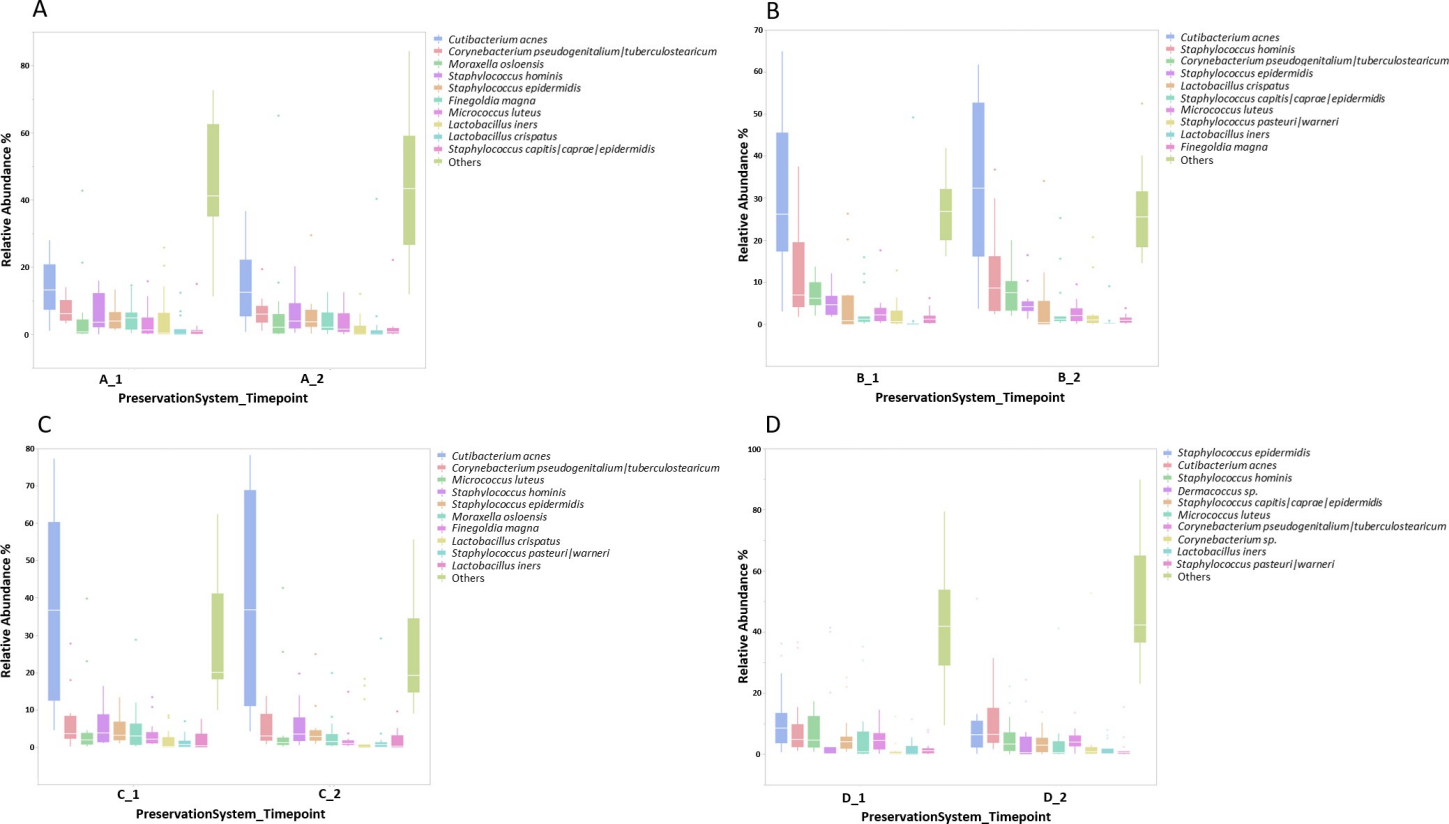
Taxonomic analysis of leg skin microbiome before and after formulation application. Species level taxonomic analysis of leg skin microbiome at baseline (T1) and following product application (T2). Panels A-D correspond to preservation systems A-D and show the top 10 most abundant species in each study.

Results of taxonomic analysis can be seen in Fig 1A–1D showing the top 10 most abundant species in each study. Figures A-D correspond to products/preservation systems A-D. In all studies the leg skin microbiome was dominated by species from the genera *Staphylococcus*, *Cutibacterium* and *Corynebacterium* with species from the genera *Moraxella*, *Micrococcus*, *Lactobacillus* and *Dermacoccus* present in different study subject populations.

Differential abundance assessment was carried out using ANCOM using a centred log ratio approach on all genera/species in the dataset. No differentially abundant species were identified between timepoints for each individual product application group.

### Alpha diversity

**Fig 2:**
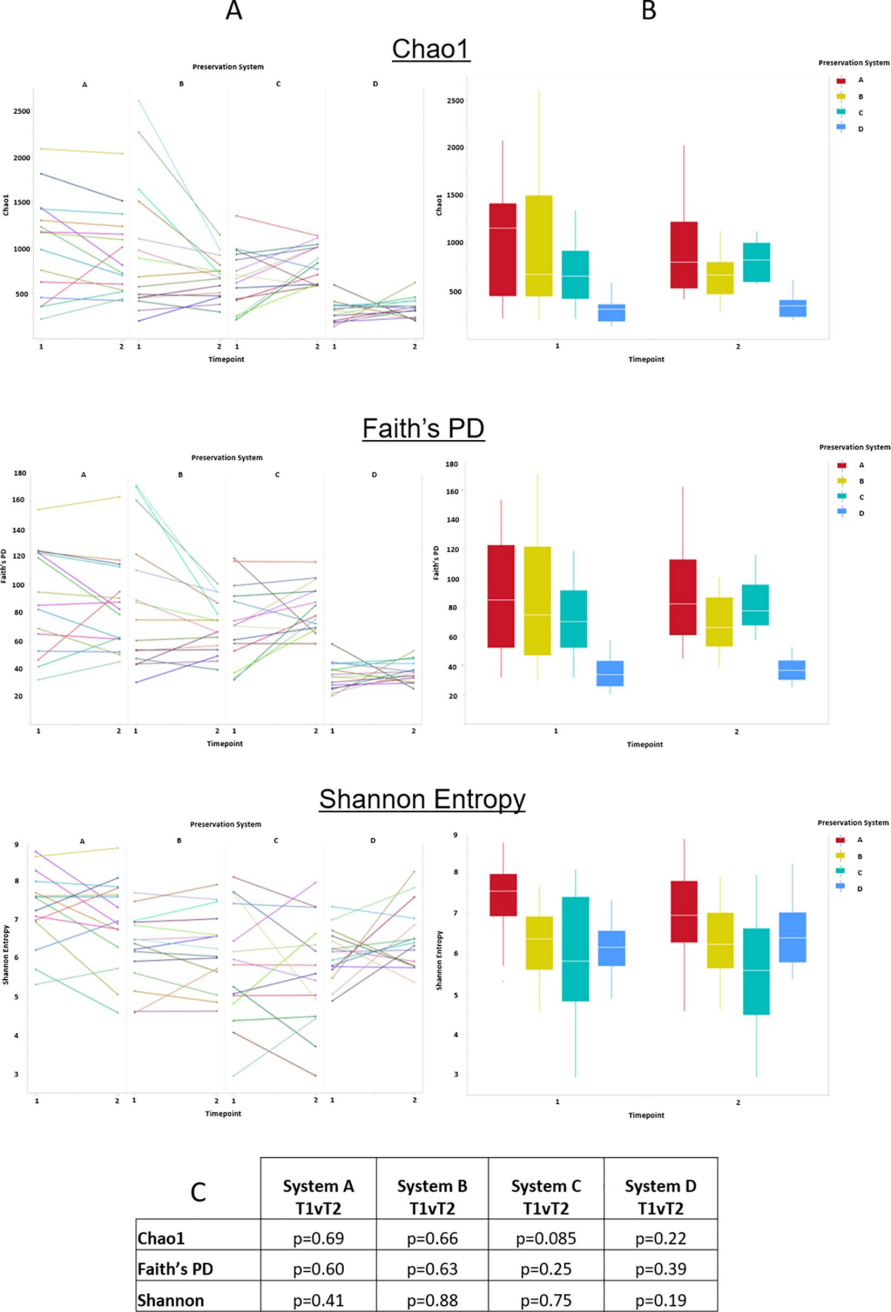
Alpha diversity analysis of leg skin microbiome before and after formulation application. A, Per sample alpha diversity assessment of the impact of the different preservation systems on the skin microbiome. B, Group alpha diversity assessment of the impact of the different preservation systems on the skin microbiome. C, Statistical analysis of alpha diversity changes between timepoints for each preservation system.

Alpha diversity analysis using 3 commonly used metrics, Chao1, Faith’s Phylogenetic Distance and Shannon Entropy were carried out to assess the impact of the different preservative systems on the leg skin microbiome following treatment. All samples were rarefied at a read count of 8000 as determined from analysis of appropriate rarefaction curves. A summary of alpha diversity analysis can be seen in Fig 2A (per subject analysis) and Fig 2B (per group analysis). For all metrics examined no significant differences were seen in alpha diversity of the leg skin microbiome following product application, Fig 2C.

### Beta diversity

**Fig 3:**
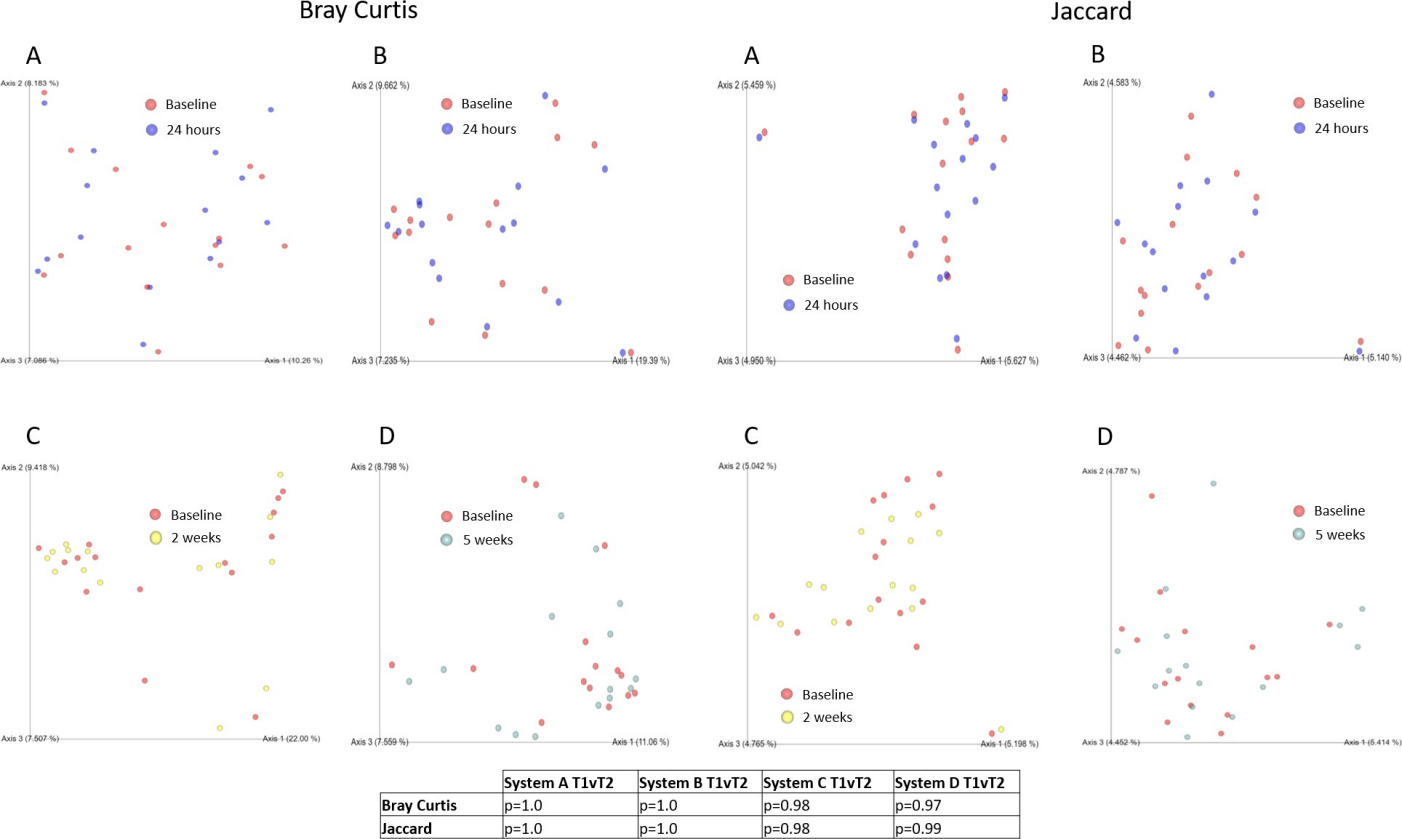
Beta diversity analysis of leg skin microbiome before and after formulation application. Analysis of the impact of cosmetic products containing different preservation systems (A-D) using Bray Curtis and Jaccard Diversity metrics. Panels A-D correspond to preservation systems A-D.

Potential changes in microbiome community structure were examined using beta diversity for all product groups. Beta diversity metrics Bray Curtis [46] and Jaccard [47] were used to determine if significant community shifts were occurring following product use. PcoA analysis of beta diversity analysis can be seen in Fig 3A–3D.

No statistical differences were identified in either beta diversity metric following product application. Additional analysis (not shown) was carried out using both weighted UniFrac and unweighted UniFrac [44] diversity analysis. Neither of these metric show statistically significant shifts in community composition following product application.

## Discussion

Antimicrobial preservation systems are widely used across a range of personal care products. They provide an essential function of ensuring that bacterial and fungal growth in cosmetic formulations is controlled to enable safe use of products by consumers. With the growing realisation of the importance of the human microbiome it has been hypothesized that the impact of preservatives may extend beyond the confines of the product formulation and may have a potentially detrimental impact on the skin microbiome. While preservative compounds have indeed been shown to be active against skin relevant bacteria *in vitro* these analyses ignore three crucial elements. Firstly, the in-use concentration of cosmetic preservatives can be drastically reduced following dilution. In the case of skin wash products this can be by up to a factor of 5-10x. At these diluted concentrations, and the limited contact time, the likelihood of antimicrobial preservatives remaining active are greatly diminished. Secondly, current *in vitro* antimicrobial tests neglect a key facet of the human microbiome namely its ability to respond to external insult and re-seed its composition from skin invaginations and glands that are protected from the formulation. The “microbiome resilience” of the community is essential in the restoration of microbiome structure and function following multiple external insults that our skin is exposed to on a daily basis. Finally, cutaneous substantivity, the persistent activity of an antimicrobial agent following application, is a key consideration [48]. While *in vitro* activity of preservative ingredients and other antimicrobial agents is obvious, their ability to bind to and remain active on skin varies considerably resulting in differences between in formulation and in use activity [49].

This work set out to examine the *in vivo* effects of cosmetic formulations containing different preservation systems on the skin microbiome in full formulation. Two different product formats (wash off and leave on) and three different use durations (1 day, 2 weeks, 5 weeks) were utilised and their impact on standard microbiome metrics was examined.

Species level taxonomic assessment revealed no statistical differences in community profile following product application. Fig 1 outlines major species identified however both these, and minor community members, remained consistent following application. Where between study comparisons was not a goal in this study it is worth noting that while leg skin microbiome samples were taken from all study populations some in-between study variation exists. Two study populations (B and C) had highly abundant levels of *C*. *acnes* where studies A and D had more balanced levels of dominant community members. No significant differences exist in the age of the study populations so it is unlikely that this variation can be explained by the varying ages of the study population as previously described [50]. Currently this variation between studies remains unexplained but it is consistent at both timepoints in both studies. It is additionally noteworthy that the UK cohort (Study D) showed less inter-subject variation in taxonomic diversity in comparison to the North American cohorts potentially worthy of further investigation focussing on skin microbiome variations based on geographical location aligned to previous analysis [51]. This reduced variation may also be explained by study D having additional exclusion criteria on subjects including exclusion of smokers and subjects who were peri- or post-menopausal, both elements that have recently been shown to impact the skin microbiome [52].

Alpha diversity analysis was used to determine the impact of the formulations on skin biodiversity. Using Chao1 (richness), Faith’s Phylogenetic Distance (phylogeny-based diversity) and Shannon (richness and abundance) diversity metrics it was shown that group alpha diversity metrics remained unchanged following product use. As outlined in Fig 2, visulisation of alpha diversity changes on a per subject basis shows that a subset of individuals in each of the product groups demonstrated a reduction in diversity there were also a number of subjects that showed an increase in diversity. In general, those subjects that started with higher than average diversity reduce, where those with lower than average diversity, increased.

Finally, beta diversity analysis was used to examine the overall impact on community structure of product application. Bray Curtis diversity and Jaccard diversity were used to examine community structure weighted towards dominant and sparce community members respectively. For both metrics, no significant changes were seen in community structure as a result of product application.

Taken together these data suggest that the different preservation systems in full formulation have minimal impact on the skin microbiome. Indeed, these results are in line with recent analyses examining the potential impact of soaps and antiseptic agents following cutaneous application, which only elicited a short-term microbiome alteration [53,54]. While additional analysis may be needed to assess the short-term impact of product application this analysis shows that the leg skin microbiome is not perturbed to a point where it is unable to recover to its baseline state following product use. This was the case for wash off products that are diluted before/during use but also in the case of a leave on lotion where dilution does not occur, and contact time is extended. Future investigations should examine the impact of preservative systems using methods including shotgun metagenomics, across multiple body sites, to facilitate strain level analysis of the skin microbiome and, if possible, include no-preservative controls, not possible here due to ethics board restrictions.

## Conclusion

Preservative systems remain an essential component of current cosmetic formulations. They provide a vital means to ensure product stability and shelf life and play a key role in consumer safety. Work presented here suggests that fully formulated cosmetics products that contain a variety of preservative systems do not have any detrimental impact on the structure or diversity of the skin microbiome for both wash off and leave on product formats.

## Supporting information

S1 Table Inclusion and exclusion criteria. (PDF)

S2 Table Formulation ingredients. (PDF)

S3 Table QIIME2 software parameters. Software parameters for QIIME2 used to process and analyse metataxonomic data.
(PDF)

S4 Table Software versions. Software versions utilised to process and analyse metataxonomic data.
(PDF)
