# Supplementary material for: In-vivo impact of common cosmetic preservative systems in full formulation on the skin microbiome
Source: PLoS One. 2021 Jul 7;16(7):e0254172. doi: 10.1371/journal.pone.0254172 (PMC8263265; doi:10.1371/journal.pone.0254172)
Supplement: S1 Table — (PDF) [file pone.0254172.s001.pdf]

S1 Table: Inclusion and Exclusion Criteria

Inclusion Criteria	Description
I1	18 to 55 years,
I2	Female,
I3	Fitzpatrick skin type I-III;
I4	BMI 18.5-39.9 (inclusive)
I5	In general good health
I6	Has intact skin on test sites – no cuts, scratches, abrasions, scars, open wounds, etc.
I7	Non-diseased skin in test sites – no eczema, psoriasis, rosacea, rashes, hives, etc.
I8	Minimal hair within test sites – must shave or clip hair 48 hours or more before each study visit with assessments
I9	Willing and able to read and sign an Informed Consent Form, comply with the study restrictions, and carry out all study procedures.
**Exclusion Criteria**	**Description**
E1	Is menopausal, post-menopausal or using any form of HRT (Study D only)
E2	Has a history of serious illness that may require regular systemic medication (e.g. thyroid dysfunction, liver dysfunction) which may influence the study outcome (in the opinion of the study doctor).
E3	History of any type of cancer (if have had cancer must be greater than 6 months remission)
E4	Has any immunological disorder (including, but not limited to, hepatitis, HIV+, AIDS, systemic lupus erythematosus, rheumatoid arthritis, hepatitis)
E5	History of any dermatological condition on the leg in adult life; e.g. eczema, psoriasis, ichthyosis, atopic dermatitis
E6	Is diabetic
E7	Use of immunosuppressive drugs; e.g. methotrexate, cyclosporine, prednisolone for the two months prior to the start of the study or if has started taking these medications during the study
E8	Use of antimicrobial drugs; e.g. penicillin, cephalosporins, tetracyclins, fusidic acid for the two months prior to the start of the study.
E9	Use of any lipid lowering medication (e.g. statins) for two months prior to the start of the study.
E10	Use of any steroidal medicine (inhaled, oral or topical)
E11	Use of any topical medication on the lower outer leg
E12	Use of systemic anti-inflammatory medication on a frequent basis (at the discretion of the medical monitor)
E13	Has suspected allergy to soap, shower gel, moisturising products or any product containing yeast as well as any alcohols (such as rubbing alcohol or isopropanol)
E14	Has excessive erythema or excoriations on the lower leg

E15	Has test sites with tattoo, scars or any other features that may affect the study procedures.
E16	Is currently pregnant or breast feeding (based on self- report only), or has given birth to or breast fed a child within the last 12 months.
E17	Use of a sunbed or sun-shower in the month prior to the start of the study, or using one during the study or planning a holiday in the sun during the study.
E18	Currently suffering from any problems with the back, hips or legs that would make sampling from the lower outer leg excessively uncomfortable for the subject or logistically difficult for the study team.
E19	Has any allergy to plasters/adhesive tape
E20	Smokers (Study D only)
E21	Participated in another clinical study on the test sites within 4 weeks of study start.
E22	Currently participating in or will begin, any other study simultaneously, at the site or another facility
E23	Is a healthcare worker (nurse, hospice care, doctor, etc.)
