# Supplementary material for: In-vivo impact of common cosmetic preservative systems in full formulation on the skin microbiome
Source: PLoS One. 2021 Jul 7;16(7):e0254172. doi: 10.1371/journal.pone.0254172 (PMC8263265; doi:10.1371/journal.pone.0254172)
Supplement: S2 Table — (PDF) [file pone.0254172.s002.pdf]

S2 Table: Formulation Ingredients

**Product A**	**Product B**	**Product C**	**Product D**
Aqua	Aqua	Aqua	Aqua
Lauric Acid	Cocamidopropyl Betaine	Cocamidopropyl Betaine	Glycerin
Hydrogenated Soybean Oil	Sodium Hydroxypropyl Starch Phosphate	Sodium Hydroxypropyl Starch Phosphate	Stearic Acid
Glycerin	Lauric Acid	Lauric Acid	Isopropyl Myristate
Glycine Soja (Soybean) Oil	Sodium Lauroyl Glycinate	Sodium Lauroyl Glycinate	Mineral Oil
Petrolatum	Sodium Lauroyl Isethionate	Sodium Lauroyl Isethionate	Glycol Stearate/Stearamide AMP
Sodium Lauroyl Glycinate	Hydrogenated Soybean Oil	Hydrogenated Soybean Oil	Glyceryl Monostearate
Sodium Cocoyl Isethionate	Helianthus Annuus Seed Oil	Helianthus Annuus Hybrid Oil	Dimethicone
Sodium Lauroamphoacetate	Glycerin	Glycerin	Peg-100 Stearate
Sodium Methyl Cocoyl Taurate	Other process and stabilising agents	Other process and stabilising agents	Petrolatum
Other process and stabilising agents	Phenoxyethanol	Glydant Plus (IPBC/DMDM)	Tapioca Starch
Glydant Plus (IPBC/DMDM)	Tetrasodium EDTA	Tetrasodium EDTA	Other process and stabilising agents
Tetrasodium EDTA	Iodopropynyl Butylcarbamate		Propylparaben
			Methylparaben
			Phenoxyethanol
