# Supplementary material for: In-vivo impact of common cosmetic preservative systems in full formulation on the skin microbiome
Source: PLoS One. 2021 Jul 7;16(7):e0254172. doi: 10.1371/journal.pone.0254172 (PMC8263265; doi:10.1371/journal.pone.0254172)
Supplement: S3 Table — Software parameters for QIIME2 used to process and analyse metataxonomic data. (PDF) [file pone.0254172.s003.pdf]

S3 Table: QIIME2 Software Parameters. Software parameters for QIIME2 used to process and analyse metataxonomic data

Software Parameter	Value
FastQC filtering min gc percentage	25
FastQC filtering max gc percentage	75
FastQC filtering min average base quality	20
DADA2 forward read trim position	21
DADA2 reverse read trim position	19
DADA2 forward read truncation position	No truncation
DADA2 reverse read truncation position	No truncation
BLAST+ penalty	-5
BLAST+ reward	4
BLAST+ gapopen	5
BLAST+ gapextend	5
