# Supplementary material for: In-vivo impact of common cosmetic preservative systems in full formulation on the skin microbiome
Source: PLoS One. 2021 Jul 7;16(7):e0254172. doi: 10.1371/journal.pone.0254172 (PMC8263265; doi:10.1371/journal.pone.0254172)
Supplement: S4 Table — Software versions utilised to process and analyse metataxonomic data. (PDF) [file pone.0254172.s004.pdf]

S4 Table: Software Versions. Software versions utilised to process and analyse metataxonomic data

**Software**	**Version**
FastQC	v0.11.5
MultiQC	1.7
QIIME2	2019.4
DADA2	QIIME2 2019.1
BLAST+	2.6.0+
scikit-learn	QIIME2 2019.1
